# A Systematic Review on the Advanced Techniques of Wearable Point-of-Care Devices and Their Futuristic Applications

**DOI:** 10.3390/diagnostics13050916

**Published:** 2023-02-28

**Authors:** Drishya Prakashan, Ramya P R, Sonu Gandhi

**Affiliations:** 1 DBT-National Institute of Animal Biotechnology (NIAB), Hyderabad 500032, Telangana, India; 2 DBT-Regional Centre for Biotechnology (RCB), Faridabad 121001, Haryana, India

**Keywords:** point-of-care testing, wearable sensors, internet of things, optical, electrochemical sensors

## Abstract

Personalized point-of-care testing (POCT) devices, such as wearable sensors, enable quick access to health monitoring without the use of complex instruments. Wearable sensors are gaining popularity owing to their ability to offer regular and continuous monitoring of physiological data by dynamic, non-invasive assessments of biomarkers in biofluids such as tear, sweat, interstitial fluid and saliva. Current advancements have concentrated on the development of optical and electrochemical wearable sensors as well as advances in non-invasive measurements of biomarkers such as metabolites, hormones and microbes. For enhanced wearability and ease of operation, microfluidic sampling, multiple sensing, and portable systems have been incorporated with materials that are flexible. Although wearable sensors show promise and improved dependability, they still require more knowledge about interaction between the target sample concentrations in blood and non-invasive biofluids. In this review, we have described the importance of wearable sensors for POCT, their design and types of these devices. Following which, we emphasize on the current breakthroughs in the application of wearable sensors in the realm of wearable integrated POCT devices. Lastly, we discuss the present obstacles and forthcoming potentials including the use of Internet of Things (IoT) for offering self-healthcare using wearable POCT.

## 1. Introduction

Regular and real-time monitoring is required for improved care of individuals suffering from chronic illnesses such as cardiovascular disease, diabetes and neurological disorders. Chronic diseases, according to the World Health Organization (WHO), account for three-quarters (75%) of all deaths worldwide and impose significant economic burdens [1,2,3,4]. POCT, a quickly progressing area in clinical testing, is evolving as a current diagnostic procedure for examination, testing and other medical applications [5,6,7,8]. The era of POCT began in 1962 with the development of a new, rapid method for measuring blood glucose levels, and it was further progressed in 1977 with the advent of a rapid pregnancy test [9,10]. POCT in clinics or hospitals gained attention in the early 1990s with compact, portable devices capable of assessing several electrolytes of patients in emergency rooms. With the focus of healthcare changing toward disease prevention and early detection, as well as chronic condition monitoring, there is an increasing demand for painless, patient-centred sensor technology [11,12,13,14,15,16]. Portable devices have proven useful in diagnosis and monitoring of various health conditions, including commercialized blood glucose monitoring. Wearable sensors (WS) with continuous monitoring capacity, on the other hand, have progressed from monitoring of generic physiological biomarkers (e.g., pressure and temperature) to much more specific purposes such as diabetes management and other diseases. The advancement of flexible, elastic and electronic technology has also allowed a wide range of wearable devices for clinical diagnostic and monitoring in the individual medical field. Although not all WS for POCT have to be flexible or stretchable, those having increased deformability and conformality offer good options for developing a revolutionary next-generation WS for POCT [17]. Moreover, the incorporation of synthetic biology into wearables may broaden the possibilities for non-invasive surveillance of physiological statuses and exposure to infections or poisons [18]. WHO’s REASSURED framework during technology development includes strategies for the manufacture of a perfect POCT device. REASSURED includes real time connectivity, ease of specimen, affordability, sensitivity, specificity, user friendly, rapid and robust, equipment free and deliverable to end users [19,20,21]. Modern WS can take high-quality measures on par with controlled medical devices. As a result, the line among customer and clinical wearable gadgets is becoming less.

First-generation WS, such as shoes, watches or headsets, were primarily aimed on biophysical measuring, such as tracking an individual’s physical activity, heart rate or temperature of body [22,23]. By the widespread adoption of first-generation wearables, attention has gradually shifted to the development of a non-invasive or minimally invasive biochemical and multifunctional monitoring device, which is the subsequent stage towards personalised health care [24]. The features of wearable systems have been altered in past years, with scientists shifting their focus away from monitoring physical exercise activity and toward tackling important challenges in healthcare applications such as diabetes treatment and surveillance systems of the elderly. To achieve these objectives, researchers have made significant investments in the fabrication of wearable systems that are sensing devices which integrate a biological recognition aspect into the operation of a sensor [25]. The constantly growing rate of a new disclosed proof-of-concept research demonstrates the possible utility of WS. Several commercially available hand-worn sensors for tracking physical activity, such as Apple Watch and Fitbit, have now become progressively more popular over the general population. A number of constant glucose tracking devices have also entered the shops (for example, Medtronic’s Guardian Real-Time and Abbott’s FreeStyle Libre). One especially appealing category of mobile health sensors is bioaffinity sensors, which use a ’bio recognition’ component for high affinity of the target sample [26,27]. The inclusion of bioaffinity components with increasing selectivity and sensitivity for the identification of disease targets will expand the WS landscape and the effect of digital health. Although several of these technologies are in clinical trials, successful commercialization has proved elusive. Significant efforts are being made to commercialise non-invasive sensors. These products, however, still need large-scale evaluations, device regulatory approvals and last marketing strategies.

Further, Internet of Things (IoT) is a potential approach for providing regular, accurate, and holistic monitoring, reducing human labour and support in medical decision making. IoT is a novel idea which permits healthcare tracking using wearable devices. It is a system of physical items that have embedded techniques for the detection and interaction with the environment, and for providing autonomous communication. WS are a prominent IoT application field that has received a lot of consideration in the previous decade, owing to the fact of inexpensive fitness applications in the market sector. Hence, this review offers a summary of the importance of WS and the various types of wearable gadgets that are currently being employed in the biomedical field (from 2018 to 2022). It also emphasizes their effectiveness in monitoring various biomolecules, as well as the applications of healthcare wearable devices for diagnostic purposes. Furthermore, existing constraints and limitations of wearables in the realm of healthcare, as well as their future prospects and the use of IoTs, are also discussed.

## 2. Wearable Point-of-Care Testing for Self-Health Care

**Scheme 1:**
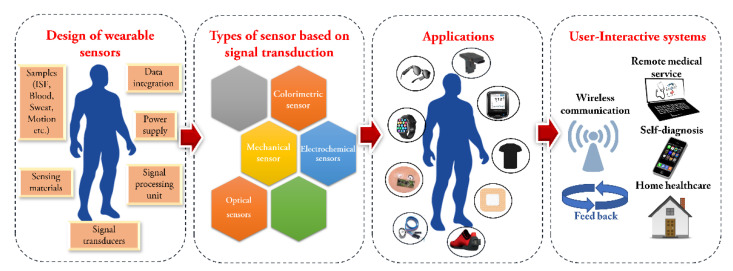
An overview of design, types and applications of wearable sensors.

Rapid advancements in sensor technology have resulted in the creation of high-performance WS for use in self-health monitoring. This expansion of WS can be attributed to a diversity of factors, such as the accessibility and ergonomics provided by advances in miniaturised electronics, the expansion of smartphones and smart devices, an increasing customer desire for awareness campaigns and the unmet need for doctors to obtain medical quality data from their patients on a continuous basis [28]. Despite this early accomplishment, there is still a strong desire to acquire even more metabolic data from the human body. WS are an outstanding alternative for medical applications because of their advantageous characteristics. They are designed, manufactured, and used in self-health care systems to aid in diagnosis of various diseases. Specific parameters, such as heart rate, pressure or temperature, can be tracked and evaluated depending on the type of sensor. Wearables provide a novel arena for monitoring of health and testing in which persons require no expertise and can monitor and evaluate their health condition intermittently or continuously on routine [29,30]. Wearable sensors can be physical, where the sensor measures the electrophysiological activity [31] (electrocardiograms, electromyograms or electroencephalograms) and physiological conditions (heart rate, temperature [32] and movement [33,34]) or biochemical, which is used to monitor body fluids by non-invasive or slightly invasive sample collection from body parts such as skin, saliva, mouth, etc., that will ultimately help in determining the state of health of the person. For continuous monitoring without any data transfer, a reader element is incorporated on the on-body gadget. The sensing component is usually colorimetric in wearable devices based on a biochemical sensor that uses optical transduction of a signal [35]. The change in colour is taken with the help of a photographic camera and transferred to a data reader, where the analysis is performed by software, that calibrates and displays the results. Scheme 1 represents the overall view of the wearable sensors (sampling, types) and their applications.

## 3. Design of Wearable Sensors

Different approaches have been made for the fabrication of WS. However, a fully integrated wearable device is still not commercially available. The ideal features of wearable POCT devices include high specificity, sensitivity, repeatability and stability, with the capability to identify multiple analytes at the same time, low cost, easy to operate and non-invasive/minimally invasive [36,37]. Hence, during the fabrication process of a WS, different parameters have to be considered, such as the method of sample collection, sensing approaches, signal processing and power supply.

### 3.1. Sampling Methods

The method of collecting a sample plays a vital part in the analysis of WS for POCT for self-use. The sampling method must be simple and easy, optimal storage conditions and its transport, no contamination and reduced intervals between sampling. The samples for these sensors include sweat, saliva, interstitial fluid (ISF), blood or wound fluids. 

#### 3.1.1. Sweat

Sweat samples are collected by sensors using absorption pads or microfluidic channels which can directly transport the sample to the sensing area where the sensor is already attached to the absorption pads [38,39]. However, this method cannot efficiently capture enough sample and the flow of sample for analysis, and chances of contamination are also high. A recent study has demonstrated the fabrication of a stretchable microfluidic platform that can collect and store the sweat in micro reservoirs, which were designed with capillary bursting valves which open at different pressures [40]. For the enhancement of the efficiency of sampling and sensing, a multimodal sweat-based device for glucose monitoring combined with feedback transdermal delivery of drug has been developed. The device works on a real time correction that is based on the measurement of temperature, pH and humidity to promote precision of sensing. Further, the feedback drug delivery contains temperature-responsive, nanoparticle-incorporated, hyaluronic acid hydrogel microneedles that allows for the controlled and accurate release of a drug in diabetic patients [41].

#### 3.1.2. Interstitial Fluid (ISF)

ISF is also an ideal biofluid for WS as they exhibit high correlation of analyte concentration with blood. The fluid can be extracted non-invasively by reverse iontophoresis device [42,43,44] or by minimally invasive microneedles that precisely disrupts the outer layer of skin with minimal pain [45].

#### 3.1.3. Blood

Blood is usually collected by pricking the tips of finger and then testing on the device. Recently, microneedles can be integrated with sensors for the regular monitoring of the analytes such as blood [46]. Microneedle patches can also be used for the sampling of blood from patients. A recent study has fabricated a one touch activated paper-based sensor with microneedle that could extract blood and produce a colorimetric detection of glucose and cholesterol concentration [47].

### 3.2. Sensor Materials

The basic requirement for the fabrication of wearable sensors includes stretchable, mechanically flexible, biodegradable and should adapt with the movement of human body. Poly (vinyl alcohol), poly(dimethylsiloxane) (PDMS), Ecoflex, polyurethane (PU), poly (ethylene terephthalate) (PET), polyester etc are some of the materials that are commonly used as sensor materials [17,18]. Similarly, several nanomaterials (organic, inorganic or hybrid) are also widely used as sensing materials [8]. Further, based on the type of sensor, the property of the material used also varies. For example, in case of sensors implanted in the body, biocompatible and biodegradable materials are used. 

### 3.3. Sensing Approaches

Wearable devices for sensing applications in sweat, blood, ISF etc have been designed by means of signal transduction methods such as electrochemical and optical signal. Colorimetry is the well-studied optical transduction approach in WS because of its minimal price, simplicity, and automated operation. Usually, in this type of sensors conventional dyes are combined with enzyme, ion indicators, or mesoporous resin beads on the surface of polymer or filter paper to create a layer for sensing [48]. The generation of novel nanomaterials with outstanding electrical and optical properties, capable to detecto all metabolic analytes (e.g., glucose, lactose) with high sensitivity and better stability are required for the fabrication of colorimetric wearables [49]. Further, wearable optical sensors which utilize fluorescence or SPR for the optical transduction method demonstrated to promote the sensitivity and specificity of the analytes [49]. Another possibility for wearable biochemical sensing is electrochemical method. Electrochemical enzymatic wearables are the most frequent used as they have unique benefits in compactness, excellent selectivity, and label-free direct monitoring. Yet, some limitations of enzymatic WS include less stability, limited sensitivity, weakness to variations in environmental parameters such as temperature, humidity and pH, and difficult manufacturing methods [50,51]. Similarly, many nonenzymatic electrochemical sensors for metabolites and electrolytes have recently been created, utilizing working electrodes made up of nanostructured materials and exhibiting remarkable sensitivity, along with limit of detection of 0.5 nM [52,53]. Recently, wearable biochemical sensors utilise radio frequency (RF) sensing devices, which works on change in RF resonance with target concentration. Because of its simple structure, reduced price, battery-free, and smartphone-communicable properties, this technique has potential for use in WS for POCT [54]. An RF based indium oxide and Pt nanoparticles sensor exhibited high responsiveness and selectivity for ethanol vapour sensing for detection of alcohol at a concentration of 200 ppm [55]. Table 1 indicated the list of analytes, its concentration and sensing component used.

### 3.4. Signal Processing Unit and Power Supply

**Figure 1:**
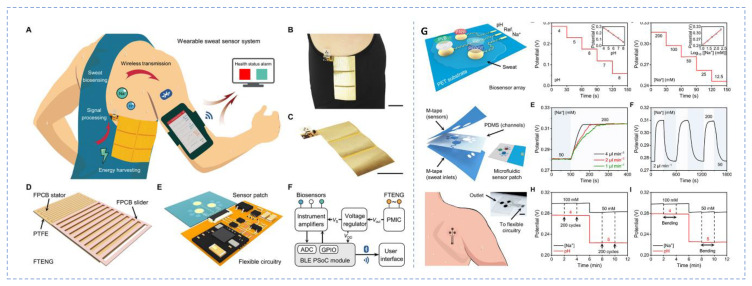
A microfluidic based wireless battery free sweat sensor (**A**). Freestanding-mode TENG (FTENG) wearable sweat sensor system (FWS^3^) for real time sweat biosensing; (**B**,**C**). Images of the sensor worn on side torso of human; (**D**). Flow chart of flexible printed circuit board based FTENG with a grating slider and an interdigital stator; (**E**). Microfluidic-based sweat analysis patch interfacing with the flexible circuitry; (**F**). Working process of the sensor; (**G**). Flexible biosensor array comprising a pH sensor and a Na^+^ sensor patterned on a flexible PET substrate and their calibration plots. (Reprinted from [34] which is an open access article distributed under the Creative Commons Attribution License).

Sensor signals must be correctly obtained and transferred to a device for processing, analysis, and display externally. For these purposes, electronic circuit boards have been established. Still, at the wearable-system level, processing, evaluation, data processing, and display of signals must be smoothly linked. Signal processing circuitry is often on the basis of integrated-circuit chips that is available in the market, but all of those components of chip are stiff, preventing the seamless incorporation at the wearable-system level. Further, the creation of self-driven sensors is critical for the progress of POCT systems because it allows for power saving in a fully integrated wearables or in the building of an independent POCT device without external power supply. Biofuel cells (BFC) have the potential to be self-driven since it can be feasibly engineered to function as self-power-driven electrochemical sensors by presenting a signal proportionate to the amount of target biomolecule in the sample [64,65]. The initial WS based on self-driven concept was developed by incorporating a BFC on a glucose biosensor device inside a contact lens for detecting the level of glucose in tears [66]. Further, the integration of biofuel cell with near field communication (NFC) enabled platform is one of the emerging means to operate a fully integrated WS [67,68]. A wireless sweat glucose sensor that operates battery free was developed on a microfluidic platform with NFC electronic module. The system was attached on the skin for the regular tracking of the concentration of glucose and lactate [69]. Similarly, a wireless, battery free, microfluidic based sweat sensor was also developed (Figure 1). Many strategies have been developed for the incorporation of multiple elements in a WS system. The issue remains in developing wearables with the needed sensitivity, selectivity, dependability, operational automation, and regular continuous evaluation. Similarly, combining all of these elements into a tiny and wearable form is a significant difficulty.

## 4. Types of Wearable Sensors

Based on a variety of aspects, including the design and utility, materials utilised, signal transduction method, nature of analyte or signal etc., WS were grouped into many types [70]. WSs can be categorised as wearable bands (watches and gloves), wearable textiles (t-shirts, socks, and shoes), wearable gear (spectacles and helmets), and sensory systems for tracking, based on their design and utility. The WS performance and wear resistance can be improved, and its usefulness can be increased, if the suitable material is available. WSs can be divided into three types based on the materials: biodegradable flexible sensors (rice-papers, nanofibers, fibroin), wearable biocompatible sensors (cellulose, chitin, alginate) and self-healing flexible sensors (hydrogel) [71]. WSs are widely divided into biophysical, biochemical, and multiplexed sensors based on the signal. Mechanical (strain, pressure, vibration, and tactile), thermal (fever), and electrophysiological (ECG, EEG, EMG and EOG), biophysical biosensors are additional subcategories of biophysical biosensors. Health-related signals such as glucose, cortisol, pH, cytokines, gas (alcohol gas sensors), hormone, nutrients, and others are picked up by biochemical sensors [72]. Biological inputs such as bacteria, cells, hormones, tissues, enzymes, chemical receptors, and other analytes that comprise antibodies, nucleic acids, and immunological agents can be detected by using either biochemical or multiplexed WS. Wearable sensors (worn on the head, neck, chest, legs, feet, arms, and fingers) are made possible by the range of transducing mechanisms that are currently accessible. A crucial element in transforming various biosensing technologies into wearable gadgets is the design of transducing mechanism. The recent advancements in colorimetric, electric, electrochemical, mechanical, and optical sensors, and the subcategories of WS are briefly described in this section [73].

### 4.1. Wearable Colorimetric Sensors

A wearable colorimetric sensing platform that included sensor patches with bromocresol green pH indicator dye in a closed headspace over the skin was studied. When basic volatile nitrogen molecules such as ammonia and amines are released from skin, the sensor spots’ colour changes [74]. The glucose oxidase (GOD)-peroxidase-o-dianisidine reagents were developed as a microfluidic chip-based wearable colorimetric sensor for detecting the glucose amount in sweat. It was discovered that the colour shift brought on by the enzymatic oxidation of o-dianisidine. The obtained linear range for sweat glucose was 0.1–0.5 mM with a detection limit of 0.03 mM [75] Another colorimetry based WSs for the detection of uric acid (UA) was fabricated by embedding poly (vinyl alcohol) microneedles that contained the uricase enzyme and catalyzed the oxidation of uric acid to produce H_2_O_2_. Polypyrrole nanoparticles (PPy NPs) with peroxidase-like activity encapsulated in the display layer generated the reaction response between H_2_O_2_ and 3,3′,5,5′- tetramethylbenzidine, that resulted in change in the colour accompanied with the amount of H_2_O_2_ formed by the uric acid oxidation [76].

### 4.2. Wearable Mechanical Sensors

High-performance wearables need strong, stretchable fibres. Fibrous materials with increased mechanical strength and tensile property are difficult to make. Ultra-robust (17.6 MPa) and extensible (700%) conducting microfibers are produced and used to make fibrous mechanical systems. The mechanical sensor is sensitive to strains with excellent resolution and a wide detection range (0.0075% to 400%). Low-frequency vibrations between 0 and 40 Hz cover most bodily tremors [77]. For the purpose of detecting mechanical deformation, wearable auxetic materials based on ionogel and metal-organic frameworks were created using 3D printing. In order to track different human body movements, the resulting auxetic sensor displayed excellent sensitivity through a change in resistance following mechanical deformation with skins [78]. A quick-resilient, hysteresis-free, vinyl hybrid silica nanoparticle (VSNPs), polyacrylamide (PAAm), and alginate double-network hydrogel-based strain sensor was created by dynamically cross-linking the PAAm network to preserve the hydrogel’s integrity. Further, the addition of VSNPs increases mechanical strength and serves as a stress buffer to release the energy. The hydrogel-based sensor’s as-prepared characteristics include strain sensitivity (also known as gauge factor) of 1.73 (up to 100% strain), a response time of 0.16 s, a very low electrical hysteresis of 2.43%, and a low LOD of 0.4% [79] Additionally. IMU (Inertial measuring unit) sensor devices are also nowadays widely used to determine and evaluate the exact force of body, angular rate and direction of the body. An IMU sensor includes a combination of three types of sensors such as Accelerometer, Gyroscope, and Magnetometer [80]. IMU are attached on different segment of the human body and measure the local motion information and hence are used for full-body motion tracking. IMU sensors integrated wrist bands often combines machine learning algorithms to analyse drinking events with 83% accuracy [81].

### 4.3. Wearable Electrochemical Sensors

Wearable electrochemical sensors provide a lot of potential for continuous, non-invasive, regular monitoring of analytes and full health evaluation. The monitoring of phenylalanine (PHE) using a wearable wristband electrochemical sensor has been described. To eliminate interferences in biofluids, the suggested electrochemical sensor is built over the screen-printed electrode (SPE) modified with a membrane made of Nafion. In order to derivatize PHE *in-situ* into an electroactive product and enable its electrochemical oxidation at the surface of SPE under alkaline circumstances, the membrane additionally contains sodium 1,2-naphthoquinone-4-sulphonate [82]. Flexible reference electrode, pH response electrode, and K^+^ selective electrode that made up the sensor were made by printing an aqueous suspension of β-CD (cyclodextrin) functionalized graphene (β-CD/RGO) on a conductive substrate using an electronic printer [83]. For constant tracking of the level of glucose in sweat with great sensitivity, a wearable electrochemical sweat sensor based on a Ni-Co MOF nanosheet covered Au/polydimethylsiloxane (PDMS) film has been developed [84]. Similarly, in another work, an intrinsically flexible, air-permeable, and body-conforming miniature liquid metal-based flexible electrochemical sensing device on fabric allowed the millimolar-level detection of glucose in sweat [85]. A sensor was developed in another study to detect a trace amount of numerous metabolites and nutrients, such as essential amino acids and vitamins, in sweat both during physical activity and at rest. It is made of graphene electrodes which can be repeatedly renewed *in-situ*. It is also functionalized with metabolite-specific antibody with molecularly imprinted polymers and redox-active reporter nanomaterials [86].

### 4.4. Wearable Optical Sensors

Li_2_ZnSiO_4_:Mn^2+^ is incorporated into stretchable elastomer-based optical fibres to create a wearable optical temperature sensor. This material can offer thermal-sensitive emissions at dual wavelengths for steady and constant ratiometric temperature monitoring that has better accuracy and repeatability [87]. A sensitive non-enzymatic fluorescence sensor for glucose detection can be made using printed circuit board substrates made of vertically aligned ZnO nanotubes (NTs). The sensor’s performance is determined by the quenching of photoluminescence (PL) in ZnO NTs treated with varied quantities of glucose. The sensor’s sensitivity is 3.5% mM^−1^ (percentage change of the PL peak intensity per mM), and its lower limit of detection (LOD) is 70 μM [88].

## 5. Application of Wearable Sensors for Self-Health Care

### 5.1. Temporary Tattoos Integrated with Sensor

**Figure 2:**
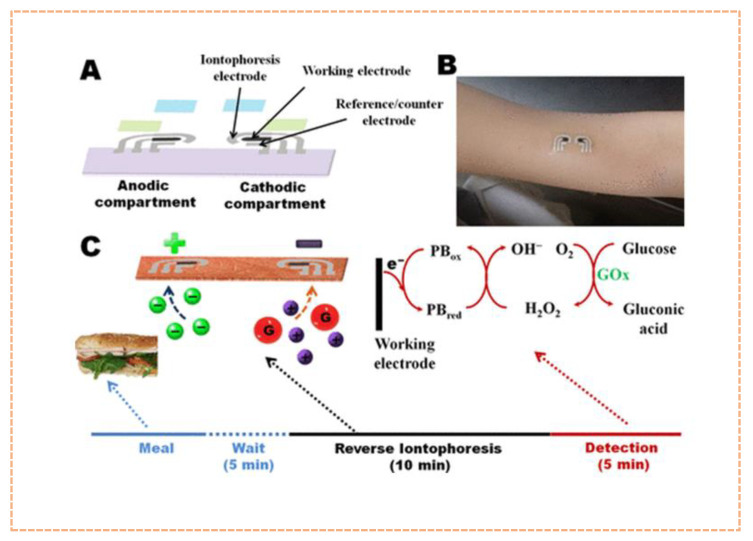
A tattoo based electrochemical sensor for glucose sensing. (**A**). The iontophoretic-sensor displaying the tattoo-based paper with Ag/AgCl and Prussian blue electrodes, insulating layer and hydrogel layer; (**B**). Image of the tattoo for glucose sensing; (**C**). Diagram representing the different processes involved in each stage of sensing. (Reprinted from [43] which is an open access article distributed under the Creative Commons Attribution License).

Temporary tattoos possess certain mechanical properties alike to body skin and may be effortlessly utilised for the real time connection in spite of repetitive mechanical twist, making them ideal for WS and POCT devices [89]. Wang’s group, in 2013 created the first tattoo-based biosensor, which was amperometric and was made by screen printing directly on the working, reference and counter electrode into a tattoo and attaching it to the skin [90]. The device was successfully tested in humans for constant monitoring of lactate in sweat during cycling. Similarly, a tattoo-based sensing system was fabricated for glucose monitoring at rest state. This was the first wearable tattoo sensor that combined reverse iontophoretic method for the collection of glucose in the ISF and an enzyme based amperometric biosensor (Figure 2) [43]. Conventional screen printing and solid contact ion selective electrode method was used for the fabrication of a tattoo that can monitor the epidermal pH level from human perspiration real time during an active physical activity [91]. Similar method was also used for the development of a skin worn sensor that could detect ammonia in sweat within a range of 10^−4^ M to 0.1 M, well within the physiological levels [92]. Tattoo-based wearable devices hold great potential for non-invasive measurement of analyte molecules such as glucose, lactate, ammonium, pH, alcohol, and salt) and may constitute the next generation of body-comfortable, wearable POCT devices. These technologies, however, exhibits certain limits. The limit of detection of tattoo-based sensors, for example, is greater than tens of mM, that is greater than the minute (mM-nM) levels of various biomolecules in human body fluids. The majority of tattoo-based sensors rely on enzyme-catalysed processes that are effortlessly influenced by temperature, humidity, and pH [93]. As a result, this parameter correction must be counted when calibrating analyte levels in bodily fluids. Furthermore, developing a tattoo with multi-sensing capabilities at the same time remains a significant difficulty. More work should be put into developing flexible electronic boards, such as signal readout and data analysing systems, as well as an interfacing technique for developing tattoo sensor devices which provide continuous, on-body analyte measurements [94].

### 5.2. Accessories Integrated with Sensor

**Figure 3:**
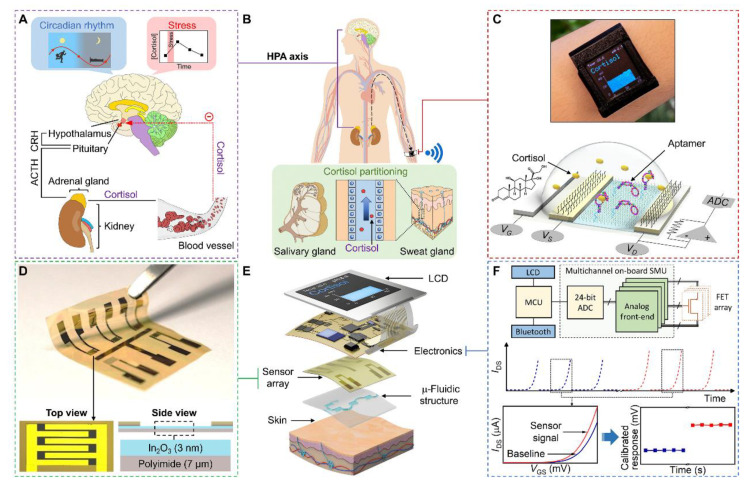
Wearable aptamer field effect transistor (FET) sensor for cortisol monitoring in sweat. (**A**). The control of level of cortisol in response to stress and circadian rhythm by hypothalamus-pituitary-adrenal axis; (**B**). Salivary and sweat glands excrete a portion of cortisol that is not bound to blood plasma proteins; (**C**). Analysis of saliva and sweat samples by the FET sensor; (**D**). Fabrication of the FET sensor with In_2_O_3_ semiconductor channels on a polyimide substrate; (**E**). Depiction of the components of the aptamer FET sensor smartwatch; (**F**). Summary of the FET sensor signal acquisition and data processing via source measurement unit (SMU) and microcontroller unit (MCU), display, and transmission (I_DS_-source-drain current; V_GS_-gate voltage) (Reprinted from [95] which is an open access article distributed under the Creative Commons Attribution License).

Recently, wearable accessories in the kind of watches, spectacles, mouth guards, and contact lenses, are intended to analyse perspiration, ISF, tears, and saliva. These accessories are incorporated with the ability for signal detection and data processing elements (such as potentiostats, microcontrollers, and bluetooth wireless communication modules) to regulate operation of system, translate analogue signals to digital signals, analyse digitalised information, and transfer data to off-body system for regular, on-body tracking. Field effect transistor based wearable smart watch-based sensor was developed for the detection cortisol in sweat and saliva samples (Figure 3) [95]. Tierney et al. fabricated the first biochemical sensor-incorporated with accessory, Glucowatches, which can continuously track acute and long-term diabetes. It included a disposable sensor, a CPU, memory for storage of data, and a liquid-crystal display. The microprocessor’s electronic circuitry regulated a reverse iontophoresis current to induce the interstitial fluid and the amperometric biosensor to detect glucose. The Glucowatches were capable of measuring and displaying levels of glucose every minute for 12 hours with precision and accuracy similar to finger prick blood glucose tests [42]. Similarly, first ever fully integrated eyeglasses capable of monitoring sweat electrolyte and metabolites was developed in 2017 [96]. The device has been fabricated by integration of an amperometric lactate sensor and a potentiometric potassium ion selective electrode at the nose bridge pads of eyeglasses. Wearable sensor for detection of salivary metabolite were fabricated by the incorporation of a printed enzymatic electrode into a mouthguard [97]. The mouthguard enzymatic sensor on the basis of an immobilised lactate oxidase and a less potential detection of the peroxide product, demonstrates good stability, sensitivity, and selectivity. Further, a sensor integrated contact lens has also been demonstrated for the in situ evaluation of glucose and lactose in tear [98,99,100,101,102]. However, the use of these contact lenses may hinder the field of vision. Researchers have now focused on the development of actual ocular contact lens to overcome this limitation. Kim et al. group demonstrated the reliable and safe operation of an ocular contact lens in an in vivo test using a live rabbit and bovine eyeball, respectively [103]. More researches are yet to be performed before commercialisation of these accessories. Furthermore, creating a fully integrated sensor device system on contact lenses capable of multiplexing for regular measurement of body analytes still persists as a significant problem.

### 5.3. Wound Dressings Integrated with Sensor

**Figure 4:**
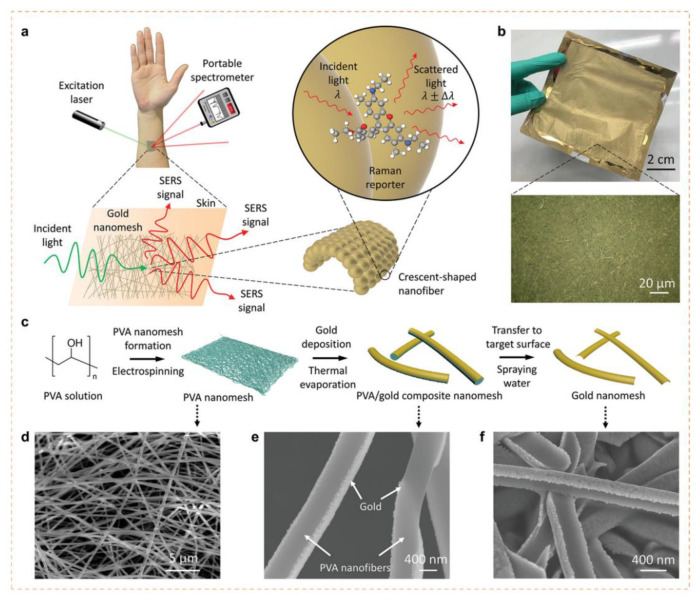
Design, characterisation and fabrication of wearable SERS sensor for detection of sweat biomarkers (**a**). Scheme representing the design and idea of wearable SERS sensor on skin; (**b**). Image of the gold nanomesh fabricated. Inset depicts the optical microscopy image of the gold nanomesh (50X); (**c**). Scheme representing the development procedure of nanomesh by electrospinning and further treatment process of the gold nanomesh; SEM micrograph of the (**d**). PVA fiber nanomesh; (**e**). Gold coated PVA fiber nanomesh; (**f**). Gold nanomesh after eliminating the PVA fibers. (Reprinted from [109] which is an open access article distributed under the Creative Commons Attribution License).

Wound healing monitoring is another key application of WS systems. Wearable sensors can monitor biological factors such as pH [104,105], temperature [106], bacterial metabolites, and sweat biomarkers (Figure 4) [107,108,109] in wound fluids. Further, these measurements can advise the patients about the healing process of wounds and properly evaluate the wound condition [110,111]. 

**Figure 5:**
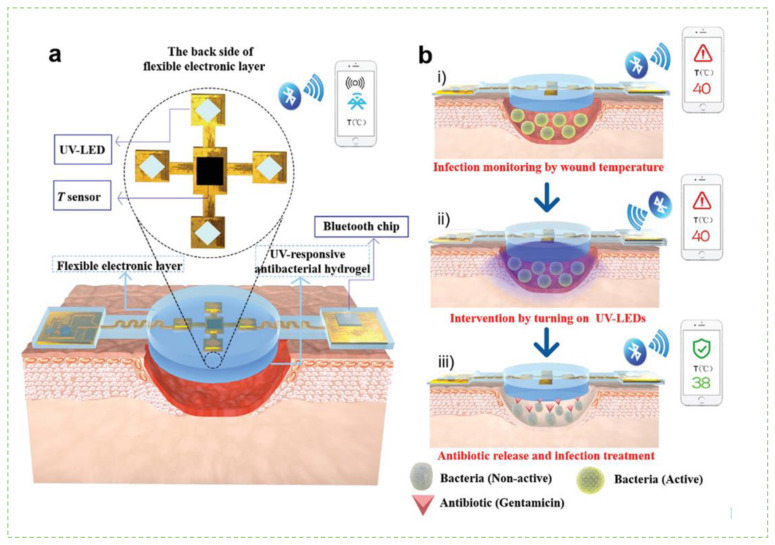
The design and the working of the smart device for wound dressings (**a**). The device consists of polydimethylsiloxane surrounded electronic layer and an antibacterial hydrogel to monitor the temperature and four UV LEDs that emit UV light to release the antibiotic from hydrogel; (**b**). Illustration of the working of the device during wound monitoring and treatment. (i). Infection is monitored by change in wound temperature; (ii). Any change is intervened by turning of UV LEDs; (iii). Antibiotics are then released onto the wound area. (Reprinted from [117] which is an open access article distributed under the Creative Commons Attribution License).

Several WS inserted in bandages/wound dressings are used to monitor analyte levels in wound fluid in real time [112]. Smart bandages with inkjet printed sensors were developed to detect variations in pH, external pressure and irregular bleeding at wound site. Further, smart bandage sensors were combined with microneedle biosensors for the screening of skin melanoma. This sensor was capable to identify the incidence of cancer biomarker tyrosinase enzyme (TYR) [113]. The tyrosinase levels dosed into the pig skin were precisely detected equally by bandage and microneedle sensors. The creation of completely integrated bandage and microneedle TYR sensors represents a potential breakthrough for melanoma screening. In addition, studies have also focused on the development of bandages capable of wound dressings and drug delivery at wound site. GelDerm is one such multifunctional dressing fabricated by Mirani group, in 2017 for the colorimetric detection of change in pH during bacterial infection and sustained release of antibiotics at the wound site [114]. Similarly, wound dressing made by incorporating a composite fibre including a core layer acting as a microheater and a hydrogel layer of cells were fabricated for the controlled release of antibiotics at wound release at wound site and vascular endothelial factor (VEGF) for promoting angiogenesis. The bandage efficiency was confirmed to improve the diabetic wound healing in murine model [115]. The smart wound bandages can hold various medications into fabrics by means of textile methods. Textile method include sensing devices that are structurally or mechanically incorporated into a textile enabling them to detect a variety of stimulants. Another sign for wound indication is the presence of uric acid. As a result, certain uric acid sensors based on smart bandages have been developed. The wearable potentiostat and the omniphobic paper based smart bandages demonstrated to wirelessly convey the condition of wound to the user or medical staff while concurrently quantifying pH and uric acid present at the injury site [116]. A polydimethylsiloxane-based temperature sensor and ultraviolet LEDs provided regular monitoring of wound-temperature and enabled the release of antibiotics from the hydrogel layer via UV irradiation (Figure 5).

In another approach, uricase enzyme paired with catalytic Prussian blue transducer, facilitated the chronoamperometric identification of the presence of uric acid at wound site with high sensitivity [118]. Table 2 represented the list of some of the WS system for POCT and Table 3 represents some of the commercially available portable sensing devices.

## 6. Challenges and Future Outlooks for Mobile and Wearable POCT Systems

Recent advancements in flexible electronics hold potential for healthcare monitoring. Much progress has been made in the integration of wearable technology, communication and data analysis technologies in order to achieve the objective of remote monitoring persons in their homes and communities [133]. Internet of Things (IoT) is a new and revolutionary topic that is gaining popularity and potential in practically every domain. Future generations of wearable IoT offer to alter the healthcare sector by seamlessly tracking individuals for tailored health and fitness data vital parameters, biological and physical activity, habits and other essential measurements that affects day-to-day life [134,135]. An IoT gateway has been built as a transitional bridge between the physical layer (sensor nodes) and the server to permit effective end-to-end communications amongst the user and the clinic for real-time monitoring. All IoT-based health systems contain a sensor layer that is involved in data collection from users by monitoring vital signs and other required signals and converting it to information that can be analysed and evaluated. Despite recent significant advances in this field, significant obstacles remain for self-driven wearables application in the biomedical field. More advances in selectivity, sensitivity, repeatability and stability of sensors, along with its mechanical durability are widely desired for regular multimodal sensing, particularly for human activity surveillances and monitoring of well-being. However, certain obstacles still exist, such as effective energy harvesting, human–device interaction and refining the measurement value and range. The integration of various powering sources, sensors, processing and testing in a non-controlled human context is critical for creating trust in these systems’ diagnostic capabilities and potential to modify outcomes [136,137]. Remote surveillance of elder persons and those taking clinical therapies will quickly necessitate the development of commercial prototypes to cover prices and discover ways of compensating for the technology and its management. Moreover, a large-scale human experiment of integrated self-driven WS is required to demonstrate their usefulness in practical applications. Interdisciplinary cooperation in the domains of material science, engineering, medicine and chemistry will be required to realise the full possibility of self-driven wearable sensing devices. Large-scale multimodal data obtained from cohort studies, combined with modern data mining technologies, could pave the way for a plethora of tailored healthcare applications [137,138]. In addition, IoT is a rising business in the technology sector, and with such a surge in usage and prospective utilisation comes a slew of security concerns. Several issues in cloud databases can degrade the IoT experience, resulting in instability and loss of trust among IoT users [139,140]. Further, developing a robust evidence foundation for the usefulness of these device systems, as well as resolving cost and compensation issues, will be critical to ensuring that WS devices live up to their potential of increasing the value of care for elder persons and people with chronic diseases.

## 7. Conclusions

Wearable sensors with novel structural designs paired with functional micro/nanomaterials enable the continuous tracking of a medical status at both physiological and biochemical levels. Wearable sensors are promising and have the potential to revolutionise therapies and diagnosis by altering the method of data collection, processing and analysis. Possible applications are numerous and are predicted to expand significantly as they get integrated into everyday wearables. WS integrated with nano-diagnostic systems have been effectively utilised for the identification of samples varying from different biomolecules such as proteins, hormones and nucleic acids) to infectious disease-causing microbes. However, more studies on the in-depth investigation of the compositions of diverse biofluids is required for identifying previously unknown biomarkers. Further, most analyte concentrations are not the same in biofluids as they are in blood, hence, establishing a good correlation between the composition of biofluids and blood chemistry is important for any future commercial applications. Moreover, the sample collection methods, sensing approaches, power source and data transmission methods must be upgraded for the fabrication of convenient wearable devices. Therefore, a better understanding of these fundamental components and analysis methods in the WS sector will soon help in the progress of the healthcare field.

## Figures and Tables

**Table 1:** The sensing ranges of different analytes in biofluids and the recognition/sensing component used for the analysis.

Sl. No.	Analyte	Biofluid	Concentration Range	Sensing/Recognition Element	Reference
1	Glucose	Interstitial fluid	36–50 μM	Non-enzymatic sensor	[56]
2	Glucose	Interstitial fluid	1.0-00 μM	Iontophoretic sensor	[43]
3	Lactate	Saliva	0.1–1.0 mM	Enzymatic sensor	[57]
4	Lactate	Sweat	1.5–100 mM	Colorimetric enzymatic sensor	[58]
5	Uric acid	Saliva	100–250 μM	Electrochemical enzymatic sensor	[59]
6	Glucose	Tear	0–50 mM	Optical sensor	[60]
7	Cl^¯^, Zn^2+^	Sweat	5.0–100 mM, 1.0–20 μM	Fluorescent sensor	[61]
8	pH	Wound fluid	6.5 to 8.5 pH	Optical sensor	[62]
9	Ca^2+^, pH	sweat, urine, and tears	1.0–0.5 mM, 4.0–7.0 pH	Electrochemical sensor	[63]

**Table 2:** List of some of the wearable sensor system for POCT of different biomolecules.

Sl No.	Form Factor/Name	Sample	Analyte	Reference
1	A Flash Glucose Monitoring System	Interstitial fluid	Glucose	[119]
2	Wrist band	Sweat	Glucose, lactate, Na^+^, K^+^	[120]
3	Wrist band	Sweat and urine	Cu, Pb, and Hg ions	[121]
4	Skin patch	Interstitial fluid	Glucose	[122]
5	Microfluidic patch	Sweat	Glucose, lactate, pH, Cl^−^	[123]
6	Microneedle patch	Interstitial fluid	Alcohol	[124]
7	Stretchable nanofiber patch	Sweat	Glucose	[125]
8	Temporary tattoo	Sweat, Interstitial fluid	Glucose and Alcohol	[126]
9	Flexible patch	Sweat	Ammonia, Lactate	[127]
10	Contact lens	Tear	Glucose	[128]
11	Bandage	Wound fluid	pH, uric acid	[129]
12	Bandage	Wound fluid	pH	[130]
13	Wound dressing	Wound fluid	Uric acid	[131]
14	Wound dressing	Wound fluid	pH	[132]

**Table 3:** List of some of the commercially available wearable sensors.

Wearable Device	Company Name	Analyte/ Signal	Description
VitalPatch^®^ Biosensor	Vital connect	ECGheart rate variability, R-R interval, respiratory rate, body temperature, skin temperature, fall detection, activity and posture	Wireless Battery operated Worn on torso 100% specificity 93.8% sensitivity
1AX Biosensor	LifeSignals	2-channel ECG, respiration rate, heart rate and temperature	Wireless Li-MnO_2_ battery 28 g in weight
FreeStyle Libre sensor	Abbott	ISF-Glucose	Continuous glucose monitoring (CGM) system consisting of a handheld reader, and a disposable sensor worn on arm
Rightest GM 300	Bionime	Blood-Glucose	Electrochemical sensor, 95% sensitivity
One Touch Ultra Link	LifeScan	Blood-Glucose	Glucose oxidase biosensor

## Data Availability

Not applicable.
